# Thyrotoxic Periodic Paralysis With Hypokalemia: A Case Study

**DOI:** 10.7759/cureus.40757

**Published:** 2023-06-21

**Authors:** Nova B Mebane, Aisha Khan, Manzoor Bevinal, Erin Davis

**Affiliations:** 1 Internal Medicine, Corpus Christi Medical Center, Corpus Christi, USA; 2 Internal Medicine, Texas College of Osteopathic Medicine, Fort Worth, USA

**Keywords:** glucocorticoid injection, thyrotoxic periodic paralysis, hypokalemia, thyrotoxicosis, hyperthyroidism

## Abstract

Thyrotoxic periodic paralysis (TPP) is a rare life-threatening condition most commonly seen in individuals between the ages of 20-40 years. It is most prevalent in Hispanic and Asian populations. Here we present a case report of a young male patient admitted to our facility with an acute onset of paralysis. He was found to have new-onset hyperthyroidism and severe hypokalemia. TPP was exacerbated by the intake of a high-carbohydrate meal as well as a steroid injection within 24 hours of symptom onset.

## Introduction

Thyrotoxic periodic paralysis (TPP) is a rare complication of thyrotoxicosis seen in young adults and is most prevalent in Asian and Hispanic populations [1]. The incidence is 2% in Asian populations and 0.1%-0.2% in North Americans. Interestingly, it is more common in males than in females; about a 26:1 difference despite the higher incidence of thyrotoxicosis in females [1]. It is theorized that testosterone can increase the activity of the sodium/potassium (Na+/K+) pump resulting in hypokalemia [2]. Furthermore, hypokalemia in TPP could be induced by the intracellular shift of potassium stimulated by triiodothyronine (T3) acting on the Na+/K+ pump. Symptoms of flaccid paralysis typically last approximately three hours to 36 hours.

Hypokalemia with periodic paralysis is a medical emergency. Up to 43.3% of patients with hypokalemic paralysis have a secondary cause with the most common cause being a complication of thyrotoxicosis [3]. Etiologies associated with TPP include Graves' disease, toxic multinodular goiter, toxic thyroid adenoma, thyroid-stimulating hormone (TSH) secreting pituitary adenomas, painless thyroiditis, and in patients taking levothyroxine [4]. The diagnostic criteria for evaluation of Graves’ disease include signs and symptoms of hyperthyroidism (tachycardia, weight loss, heat intolerance, tremor, etc.), ophthalmopathy, and goiter. It is important to acknowledge that one does not have to have goiter or ophthalmopathy to be diagnosed with Graves’ disease.

Typically, TPP manifests as transient episodes of lower extremity weakness ranging from weakness to flaccid paralysis. Generally, sensory functions including bowel and bladder functions remain intact. In order to consider TPP as a primary diagnosis, the patient has to have high levels of thyroid hormone. A similar disorder showing symptoms of paralysis is known as Familial Hypokalemic Periodic Paralysis (HKPP). This is a rare autosomal dominant channelopathy with a defect in calcium channels resulting in muscle paralysis, but there is no hyperthyroidism in these individuals [5]. Thus, it is important to consider obtaining a TSH to assess thyroid function when a patient has hypokalemia and periodic paralysis with no obvious initial cause.

The treatment of TPP includes the acute management of thyrotoxicosis with beta-blockers, antithyroid therapy, and the cautious correction of hypokalemia. Early diagnosis and treatment are aimed at correcting muscular symptoms so as to prevent the paralysis of respiratory muscles.

## Case presentation

A 25-year-old male of Hispanic and Caucasian descent with no significant past medical history presented to our emergency department from an outlying facility for acute onset of paralysis starting in bilateral lower extremities. He reported receiving a recent intraarticular corticosteroid injection to the right knee less than 24 hours prior to the onset of his symptoms. He consumed a high carbohydrate meal within five hours prior to symptom onset. Bilateral lower extremity weakness progressed to his trunk and bilateral upper extremities. At the time of our evaluation, the patient was asymptomatic due to intravenous correction of hypokalemia at the outlying facility.

At the outlying facility, vital signs were normal. Physical exam was remarkable for paralysis of his extremities. The sensation was intact. Initial labs were remarkable for severe hypokalemia of 1.9 mmol/L (rr 3.6-5.2) and TSH less than 0.01 (rr 0.42-5.47). The patient was transferred to our facility for further evaluation.

**Figure 1:**
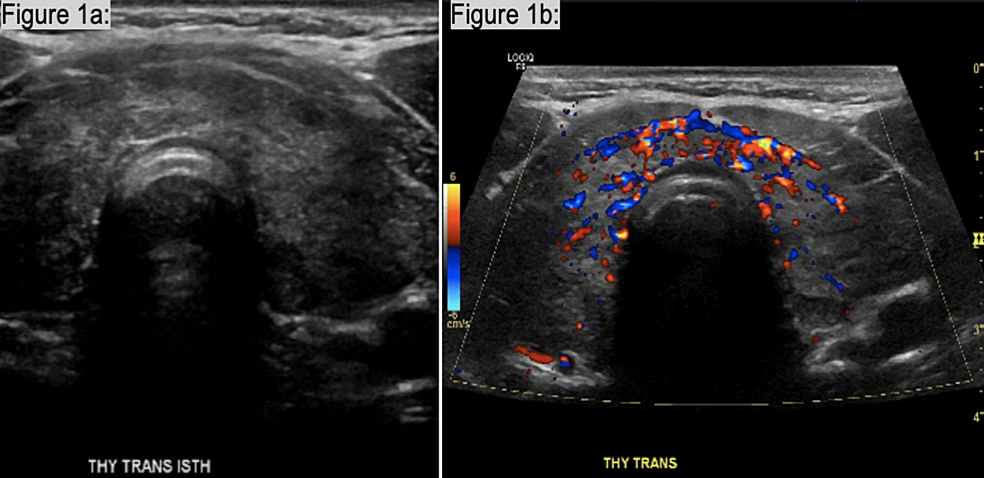
Thyroid transverse views of the isthmus. (a) Heterogenous regions of thyroid with no apparent lesions. (b) Color Doppler showing increased vascularity in the thyroid.

Pertinent lab values during his hospital admission include elevated free thyroxine (T4) level at 3.73 ng/dL (rr 0.59-1.17), elevated TSH binding inhibitory immunoglobulin 8.60 IU/L (rr 0.00-1.75), glycated hemoglobin 5.6 (rr 4.5-6.2), urine glucose 1,000 mg/dL, urine ketones 5 mg/dL, and urine blood 2+. Imaging reported junctional rhythm with 48 ventricular rates on EKG, no radiographic evidence of acute cardiopulmonary process on Chest X-ray one-view, and no evidence of acute intracranial abnormality or hemorrhage on computed tomography (CT) head without contrast. A thyroid ultrasound showed heterogeneous vascular thyroid with no discrete mass or lesion (Figures 1a, 1b).

A tele-medicine neurologist evaluated the patient and recommended a cardiology consult given the potential for arrhythmias, low threshold for cerebral spinal fluid analysis as well as total neuroaxis magnetic resonance imaging (MRI) with and without gadolinium, and onsite neurology evaluation to conduct electrodiagnostic studies. However, due to the patient not having local residence, he was discharged once sufficient evaluation was finished and he was back to baseline in motor and sensory function. He was instructed to establish care with a primary care provider in his hometown and seek a referral to an endocrinologist for proper management of his hyperthyroidism. He was discharged with methimazole 10mg daily and propranolol 10mg twice a day.

The patient was followed up three weeks later and denied major features of hyperthyroidism including anxiety, tremors, muscle weakness, unprovoked sweating, and heart palpitations. However, he did report lightheadedness shortly after taking his second dose of propranolol in the evenings. He was instructed to decrease propranolol to 10mg daily. He stated that a primary care physician appointment was scheduled within the week in his hometown; thus, no further follow up was necessary from our facility.

## Discussion

We diagnosed this patient with TPP as opposed to familial HKPP. The latter tends to have an onset of paralysis less than age 16, have greater than 24 hours of paralysis, have a familial history of paralysis, and may present with hypomagnesemia and/or hypophosphatemia [6]. Our patient described in this study had specific features associated with TPP which included being an adult male, laboratory values indicative of hyperthyroidism, having no immediate family history of paralysis, and being symptomatic for no more than six hours.

We cannot determine whether this patient had Graves’ disease associated with TPP. Unfortunately, we were unable to obtain a thyroid-stimulating immunoglobulin (TSI) assay, which is 96% sensitive and 99% specific for Graves’ disease [7]. However, we were able to obtain a TSH receptor binding inhibitory immunoglobulin assay (TBIAb) which has an incidence of 70.9% (untreated Graves’ disease), 53.1% (currently being treated for Graves’ disease), and 19.2% (in remission from Graves’ disease) [8].

Thyroid hormone increases sensitivity to catecholamines and sensitizes beta-2 adrenergic receptors. This results in the stimulation of Na+/K+ ATPase channels in the liver and skeletal muscle allowing for potassium to move into cells [9]. The thyroid hormone also upregulates the potassium leak channel, decreasing the threshold for an action potential. A genetic analysis would have provided significant information towards a possible hereditary link predisposing him to TPP by assessing CACNA1S (gene for voltage-gated calcium channels), SCN4A (gene for sodium channels), and KCNJ18 (gene for inwardly rectifying potassium channels and units for sodium-potassium pump) [10]. The resulting hypokalemia due to these channelopathies may induce cardiac arrhythmia, muscle cramps, paralysis, paresthesias, vomiting, and glucose intolerance. Individuals susceptible to TPP may have a channelopathy involving a loss-of-function mutation of the Kir2.6 channel, an inwardly rectifying K+ channel, which is usually stimulated by thyroid hormones. This loss of function may predispose someone to periodic paralysis by disrupting K+ regulation, resulting in the inactivation of muscle sarcolemmal sodium channels and subsequent paralysis [11].

Any stimulus that can cause hypokalemia could exacerbate or cause TPP in someone who has uncontrolled hyperthyroidism. Some reported precipitating factors of TPP include consumption of a high carbohydrate meal, corticosteroids, stress, a high sodium diet, alcohol, rest after exercise, and exposure to cold temperatures [12]. However, a study by Chang et al. showed only 34% of patients had a precipitating factor. Thus, no additional factors besides hyperthyroidism are necessary in order for TPP to occur [12]. The patient, in this case, study reported having a high carbohydrate meal shortly before the onset of symptoms, and his first intra-articular glucocorticoid injection was within 24 hours of symptom onset. We conclude that the combination of those known precipitating factors in addition to undiagnosed hyperthyroidism resulted in this patient’s paralysis. The rise in serum glucose levels after a meal causes the release of insulin from pancreatic β cells. Insulin increases Na+/K+ ATPase channel activity which shifts glucose and potassium into cells, thus potentiating hypokalemia. From the current review of the literature, there are at least 13 cases of TPP associated with glucocorticoid use [13-16]. Glucocorticoid treatment for Graves’ ophthalmopathy or thyroid storm may precipitate TPP via a negative feedback mechanism [16]. Corticosteroids upregulate Na+/K+ ATPase channels and mediate the release of glucose into extracellular space. An increase in serum glucose then stimulates insulin secretion, resulting in a shift of potassium from the intracellular to extracellular space.

In patients presenting with paralysis in the setting of low TSH and hypokalemia, it is important to replete potassium while monitoring for rebound hyperkalemia or hyperphosphatemia. Beta-blockers decrease stimulation of the Na+/K+ ATPase pump, thereby reducing the influx of potassium. Once TPP has been diagnosed, patients should have a discussion with their doctor regarding ablative therapy. Lastly, factors that may induce TPP should be avoided, including medications that cause hypokalemia.

## Conclusions

TPP is a rare condition according to prevalence. There are few published cases of glucocorticoid administration prior to TPP, thus the possibility that glucocorticoids may precipitate TPP requires further investigation. An interdisciplinary approach including but not limited to the subspecialties of cardiology, neurology, and endocrinology should be consulted when a patient presents with severe features of TPP.

Many seemingly healthy young adults can have irregular primary care follow up and hyperthyroidism can easily go undiagnosed. Obtain TSH and free T4 levels when a young male presents with paralysis and no obvious cause. 
